# Oncological safety of portal vein embolization without prior tumour clearance in the future liver remnant followed by one-stage hepatectomy for bilateral colorectal liver metastases

**DOI:** 10.1093/bjs/znaf198

**Published:** 2025-09-22

**Authors:** Tim Reese, Dennis Björk, Anne M H Longva, Kristian S Kiim, Maximilian Evers, Peter N Larsen, Nicolai A Schultz, Bård I Røsok, Ulrik Carling, Fredrik Holmquist, Gert Lindell, Per Sandström, Jörg Böcker, Stefan Gilg, Jennie Engstrand, Christian Sturesson, Karl J Oldhafer, Bergthor Björnsson, Ernesto Sparrelid

**Affiliations:** Division of Surgery and Oncology, Department of Clinical Science, Intervention, and Technology, Karolinska Institutet, Karolinska University Hospital, Stockholm, Sweden; Department of Surgery, Department of Biomedical and Clinical Sciences, Linköping University, Linköping, Sweden; Department of Hepatopancreatobiliary Surgery, Oslo University Hospital, Oslo, Norway; Department of Surgical Gastroenterology and Transplantation, Rigshospitalet, University of Copenhagen, Copenhagen, Denmark; Department of Surgery, Division of Hepatobiliary and Pancreatic Surgery, Asklepios Hospital Barmbek, Hamburg, Germany; Department of Surgical Gastroenterology and Transplantation, Rigshospitalet, University of Copenhagen, Copenhagen, Denmark; Department of Surgical Gastroenterology and Transplantation, Rigshospitalet, University of Copenhagen, Copenhagen, Denmark; Department of Hepatopancreatobiliary Surgery, Oslo University Hospital, Oslo, Norway; Department of Radiology, Rigshospitalet, Oslo University Hospital, Oslo, Norway; Department of Surgery, Skåne University Hospital, Lund, Sweden; Department of Surgery, Skåne University Hospital, Lund, Sweden; Department of Surgery, Department of Biomedical and Clinical Sciences, Linköping University, Linköping, Sweden; Department of Surgery, Division of Hepatobiliary and Pancreatic Surgery, Asklepios Hospital Barmbek, Hamburg, Germany; Division of Surgery and Oncology, Department of Clinical Science, Intervention, and Technology, Karolinska Institutet, Karolinska University Hospital, Stockholm, Sweden; Division of Surgery and Oncology, Department of Clinical Science, Intervention, and Technology, Karolinska Institutet, Karolinska University Hospital, Stockholm, Sweden; Division of Surgery and Oncology, Department of Clinical Science, Intervention, and Technology, Karolinska Institutet, Karolinska University Hospital, Stockholm, Sweden; Department of Surgery, Division of Hepatobiliary and Pancreatic Surgery, Asklepios Hospital Barmbek, Hamburg, Germany; Department of Surgery, Department of Biomedical and Clinical Sciences, Linköping University, Linköping, Sweden; Division of Surgery and Oncology, Department of Clinical Science, Intervention, and Technology, Karolinska Institutet, Karolinska University Hospital, Stockholm, Sweden

## Abstract

**Background:**

Upfront portal vein embolization (PVE) without prior future liver remnant (FLR) clearing followed by a one-stage hepatectomy (OSH) for bilateral colorectal liver metastases (CRLM) can reduce the surgical burden of a two-stage approach, but oncological safety is not well described and comparisons with alternative two-stage procedures are lacking.

**Methods:**

A retrospective cohort of patients with bilateral CRLM and tumour in the FLR, undergoing liver resection between 2013 and 2021, was studied. The patients were divided into three groups: patients who underwent PVE with no prior tumour clearance in the FLR followed by an OSH (PVE-OSH); patients who underwent tumour clearance in the FLR followed by PVE (TSH-PVE; where TSH stands for two-stage hepatectomy); and patients who underwent associating liver partition and portal vein ligation for staged hepatectomy (ALPPS).

**Results:**

In total, 302 patients with bilateral CRLM were included, of whom 127 underwent PVE-OSH, 61 underwent TSH-PVE, and 114 underwent ALPPS. Except for age and Eastern Cooperative Oncology Group (ECOG) Performance Status, all baseline characteristics were comparable. The most rapid hypertrophy was experienced by ALPPS patients, followed by PVE-OSH patients. Successful resection could not be performed in 11% of PVE-OSH patients, 21% of TSH-PVE patients, and 4% of ALPPS patients (*P* < 0.001). During major resection, 23% of TSH-PVE patients required additional FLR resection/ablation and the median time from first intervention to major resection was 9 (interquartile range (i.q.r.) 7–13) weeks, compared with 6 (i.q.r. 5–8) weeks for PVE-OSH patients and 1 (i.q.r. 1–3) week for ALPPS patients (*P* < 0.001). Postoperative outcomes were comparable regarding liver failure, mortality, and overall survival. Multivariable regression analysis for liver recurrence identified the number of metastases (HR 1.04 (95% c.i. 1.00 to 1.07); *P* = 0.025) and ALPPS (HR 1.64 (95% c.i. 1.00 to 2.68); *P* = 0.048) as independent risk factors.

**Conclusion:**

PVE-OSH can be performed safely for patients with a limited tumour burden in the FLR, thereby obviating the need for two-stage procedures.

## Teaser

For patients with bilateral colorectal liver metastases, portal vein embolization with no prior tumour clearance in the future liver remnant (FLR) followed by a one-stage hepatectomy can be safely performed, avoiding the need for two-stage procedures.

## Introduction

The management of resectable bilateral colorectal liver metastases (CRLM) presents a considerable challenge, requiring a multidisciplinary approach. When metastases are unresectable using a one-stage procedure, major hepatectomy is required, and assessing the volume of the future liver remnant (FLR) is crucial in this situation^1,2^. If the FLR is insufficient, a hypertrophy-inducing procedure is necessary to execute a safe resection. Considering tumour size and location of the metastasis, potential vascular infiltration, initial FLR, and co-morbidities, the surgical team formulates a resection plan, considering portal vein embolization (PVE) (±hepatic vein embolization), associating liver partition and portal vein ligation for staged hepatectomy (ALPPS), or classical two-stage hepatectomy (TSH). PVE is the most common hypertrophy procedure and the current ‘gold standard’, which has been shown to be a safe and effective intervention to achieve the required FLR^2^, even in two-stage procedures^3–5^.

Research has shown that liver hypertrophy can contribute to tumour progression^6,7^, because growth hormones also promote tumour growth^8^. A few studies have examined the impact of PVE on tumour growth in the FLR^9–11^. They suggest that chemotherapy reduces the risk of progression during hypertrophy. This opens up an alternative approach for bilateral CRLM, which eliminates the need for tumour clearing of the FLR before hypertrophy and therefore the need for a two-stage procedure. In this method, patients with bilateral CRLM undergo an upfront PVE and, once the required FLR is achieved, a one-stage hepatectomy (OSH) with major hepatectomy of the embolized lobe is performed, including clearance of the FLR in a one-stage procedure^9^. However, there are no comparative studies available to assess complications, recurrence, or survival for this strategy. Hence, the aim of this study was to investigate whether PVE-OSH (see the Surgical approaches section for a full description) has the same or better outcomes in terms of liver recurrence-free survival and complications compared with those of other implemented procedures.

## Methods

### Patient selection

For this retrospective study (study interval from January 2013 to December 2021), all patients with bilateral CRLM, with metastases in the FLR, and who were surgically treated with regenerative liver surgery were included from six liver surgery centres: Karolinska University Hospital, Stockholm, Sweden; Linköping University Hospital, Linköping, Sweden; Skåne University Hospital, Lund, Sweden; Oslo University Hospital, Oslo, Norway; Rigshospitalet, Copenhagen, Denmark; and Asklepios Hospital Barmbek, Hamburg, Germany. Ethical approvals were obtained from the local ethical committees. Patients who underwent intraoperative portal vein ligation (other than ALPPS) or classical TSH without PVE or who had no metastases in the FLR were excluded; patients who underwent hepatic vein embolizations (simultaneous or sequential) were also excluded.

### PVE

In most patients, access through percutaneous puncture of the ipsilateral portal vein was utilized. For embolization, glue-based material or particles, sometimes in addition to coils and plugs, were used. Although there was no standardized approach within the six centres, each centre has previously published their approach in detail^12–16^.

### Surgical approaches

**Fig. 1:**
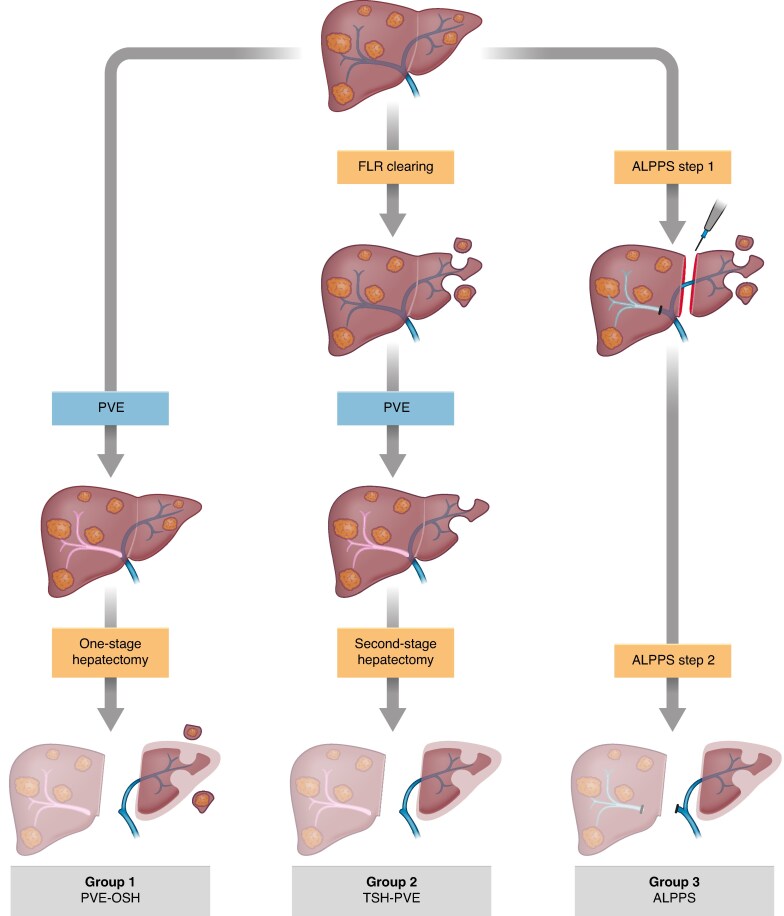
Three groups for the treatment of bilateral CRLM with tumour in the FLR Blue boxes indicate an intervention and orange boxes indicate an operation. CRLM, colorectal liver metastases; FLR, future liver remnant; PVE, portal vein embolization; PVE-OSH, PVE with no prior tumour clearance in the FLR followed by a one-stage hepatectomy; TSH-PVE, tumour clearance in the FLR followed by PVE (where TSH stands for two-stage hepatectomy); ALPPS, associating liver partition and portal vein ligation for staged hepatectomy.

The patients were divided into three groups based on the surgical approach that was used (*Fig. 1*). The PVE-OSH group included patients who underwent PVE as the first intervention, after hypertrophy of the tumour bearing FLR a one-stage procedure was performed with simultaneous local resections in the FLR and major hepatectomy of the embolized liver lobe. The TSH-PVE group included patients who underwent local liver resection in the FLR, followed by PVE; after hypertrophy of the tumour-free FLR, major resection of the embolized liver lobe was performed. The ALPPS group included patients who underwent local liver resection in the FLR with portal vein ligation and parenchymal transection in the future resection line; after hypertrophy of the tumour-free FLR, the ligated lobe was resected during the second stage. The selection of the approach for each patient was determined by the institution, as well as by the algorithms employed by the institution.

### Volumetric analysis

Each centre performed the volumetric analysis based on local routine using preoperative CT or MRI. The FLR was calculated manually or semi-automatically by outlining the future liver segments on axial planes, with remnants such as cysts, portal branches, and bile ducts excluded. The total estimated liver volume (TELV) was calculated based on the body surface area^17^. The standardized future liver remnant (sFLR) was then calculated based on the TELV^18^. Volumetric analysis was performed before the first intervention (baseline FLR) and before the major liver resection (pre-resection FLR).

### Clinical data and definitions

Patient performance status was recorded according to the Eastern Cooperative Oncology Group Performance Status Scale (ECOG PSS)^19^. Synchronous CRLM were defined according to the European-African Hepato-Pancreato-Biliary Association (E-AHPBA) synchronous colorectal cancer and liver metastases consensus guidelines^20^, with synchronous defined as the detection of metastases before or at the time of presentation of the primary tumour. Chemotherapy regimens were grouped according to the number of agents administered (single, double, or triple). The response was evaluated using the Response Evaluation Criteria in Solid Tumours (RECIST) criteria^21^.

The hypertrophy time was defined as weeks from the first hypertrophy intervention (PVE or ALPPS step 1) to the date of preoperative volumetry. Absolute growth (ml) and degree of hypertrophy (DH) (%) were measures of the volume that the liver gained (pre-resection FLR − baseline FLR). The kinetic growth rate (%/week) was calculated as follows: DH/hypertrophy time (weeks), showing the percentage that the FLR increased per week^22^.

The term ‘rescue ALPPS’ was defined as the transition from PVE to a surgical two-stage procedure involving parenchymal transection and local resections as the first stage, followed by major resection as the second stage^23^. The rescue-ALPPS patients were analysed as intention-to-treat and stayed in the initial group. Complications were classified using the Clavien–Dindo classification^24,25^. Post-hepatectomy liver failure (PHLF)^26^ and bile leak^27^ were graded according to the International Study Group of Liver Surgery (ISGLS).

### Statistical analysis

Continuous variables are reported as median (interquartile range) and were compared using the Kruskal–Wallis test. Categorical variables are reported as *n* (%) and were compared using Pearson’s chi-squared test and Fisher’s exact test. For all tests, *P* < 0.050 was considered statistically significant. Backward stepwise Cox regression analysis was performed to identify factors associated with liver recurrence-free survival. Parameters that suggested a higher risk of liver recurrence in the univariable analysis (*P* < 0.200) and variables with previously described clinical impact on the outcome (even if *P* > 0.200) were included in the analysis. The Kaplan–Meier method was used to analyse liver recurrence-free survival, disease-free survival, and overall survival. Survival was calculated from the date of major resection or first intervention to death or last follow-up if alive. Liver recurrence-free survival and disease-free survival were estimated from the date of major resection to the date of first liver or overall recurrence. Patients who were lost to follow-up or whose follow-up was terminated were censored at the last date that they were known to be alive. Differences were compared using the log rank test. Statistical analysis was performed using SPSS^®^ (IBM, Armonk, NY, USA; version 29.0.1.0).

## Results

### Patient and tumour characteristics

During the study interval, 302 patients were included in the study (*Fig. S1*) and patients were divided into the three resection strategy groups (*Fig. 1*). The cohort consisted of 127 PVE-OSH patients, 61 TSH-PVE patients, and 114 ALPPS patients. Age was significantly higher in the PVE-OSH group and the ALPPS group had significantly more ECOG PSS grade 1 patients (*Table 1*). Regarding preoperative chemotherapy, no differences were seen; however, the time from end of chemotherapy to the first intervention was longest for TSH-PVE patients. The total number of metastases was highest in the TSH-PVE group and the diameter of the largest metastases was largest in the ALPPS group. There were also significantly more metastases in the FLR in the TSH-PVE group (*Table 1*). The proportion of patients who were dropouts during the hypertrophy process was highest (21%) for the TSH-PVE group, followed by the PVE-OSH group (11%) and the ALPPS group (4%) (*P* < 0.001). Tumour progression was the most common reason for not proceeding with the resection in all three groups. A detailed description of the progression type and the reasons for not proceeding is shown in *Table S1*.

**Table 1:** Baseline patient characteristics

	PVE-OSH (*n* = 127)	TSH-PVE (*n* = 61)	ALPPS (*n* = 114)	*P*
**Sex**				
Male	88 (69.3)	38 (62.3)	76 (66.7)	0.633
Female	39 (30.7)	23 (37.7)	38 (33.3)	–
Age (years), median (i.q.r.)	65 (57–74)	61 (53–68)	61 (53–69)	0.008*
Diabetes mellitus	17 (13.4)	7 (11.5)	12 (10.5)	0.786
ECOG grade 0	83 (65.4)	41 (67.2)	46 (40.4)	<0.001*
ECOG grade 1	39 (30.7)	17 (27.9)	64 (56.1)	–
ECOG grade 2	5 (3.9)	3 (4.9)	4 (3.5)	–
**Location of primary tumour**				
Right colon	33 (26.0)	14 (23.0)	23 (20.2)	0.629
Left colon	46 (36.2)	28 (45.9)	49 (43.0)	–
Rectum	48 (37.8)	19 (31.1)	42 (36.8)	–
**Time of diagnosis and strategy**				
Metachronous	13 (10.2)	2 (3.3)	14 (12.3)	0.149
Synchronous	114 (89.8)	59 (96.7)	100 (87.7)	–
Liver first (synchronous only)	62 (48.8)	23 (37.7)	51 (44.7)	0.151
Primary first (synchronous only)	52 (40.9)	36 (59.0)	49 (43.0)	–
*KRAS* mutation (51% missing data)	25 (34.7)	11 (44.0)	18 (38.3)	0.705
*BRAF* mutation (55% missing data)	6 (8.7)	1 (4.8)	4 (8.9)	0.826
**Chemotherapy (13 missing data)**				
No neoadjuvant chemotherapy	3 (2.4)	1 (1.6)	6 (5.3)	0.300
Single	3 (2.4)	0 (0.0)	1 (0.9)	–
Double	105 (82.7)	54 (88.5)	80 (70.2)	–
Triple	13 (10.2)	6 (9.8)	17 (14.9)	–
Chemotherapy with antibody	48 (37.8)	23 (37.7)	47 (41.2)	0.365
Number of cycles of chemotherapy, median (i.q.r.)	6 (4–8)	6 (4–8)	6 (4–8)	0.970
Last cycle of chemotherapy to major resection (weeks), median (i.q.r.)	10 (7–15)	18 (12–22)	9 (7–15)	<0.001*
**Response**				
Regress	89 (71.2)	47 (82.5)	65 (78.3)	0.474
Stable	25 (20.0)	7 (12.3)	14 (16.9)	–
Progress	11 (8.8)	3 (5.3)	4 (4.8)	–
Number of lesions, median (i.q.r.)	8 (6–12)	10 (7–16)	9 (6–10)	0.048*
Largest lesion diameter (mm), median (i.q.r.)	25 (17–53)	29 (20–46)	40 (25–55)	0.029*
Number of lesions in the FLR, median (i.q.r.)	2 (1–3)	3 (2–5)	2 (1–3)	<0.001*
Largest lesion in the FLR(mm) , median (i.q.r.)	12 (7–19)	15 (10–21)	14 (10–24)	0.093
No resection	14 (11.0)	13 (21.3)	4 (3.5)	<0.001*

Values are *n* (%) unless otherwise indicated. *Statistically significant. PVE-OSH, portal vein embolization with no prior tumour clearance in the future liver remnant followed by a one-stage hepatectomy; TSH-PVE, tumour clearance in the future liver remnant followed by portal vein embolization (where TSH stands for two-stage hepatectomy); ALPPS, associating liver partition and portal vein ligation for staged hepatectomy; i.q.r., interquartile range; ECOG, Eastern Cooperative Oncology Group Performance Status Scale; FLR, future liver remnant.

### Hypertrophy

The TELV, baseline sFLR, pre-resection sFLR, and DH demonstrated comparable values across the groups (*Table S2*). However, the most rapid hypertrophy was experienced by ALPPS patients and, consequently, the highest KGR was experienced by ALPPS patients. In the PVE-OSH group, 17 patients (15%) experienced failure of hypertrophy, necessitating a change of strategy to rescue ALPPS, compared with 12 patients (35%) in the TSH-PVE group. In contrast, six patients (5%) in the ALPPS group required PVE between stages due to inadequate hypertrophy.

### FLR clearing

For tumour clearance in the FLR, see *Table 2*. Notably, 19% of the TSH-PVE group underwent at least five local resections in the FLR, which was the highest rate among the groups. Vanishing lesions were more prevalent in the PVE-OSH group (*P* = 0.027). The complication rates after FLR clearing were comparable between the TSH-PVE group and the ALPPS group, and it is noteworthy that no complications arose from PVE. Complications, operating time, and intraoperative bleeding in the PVE-OSH group are shown for patients who required rescue ALPPS.

**Table 2:** FLR clearing (clearing performed simultaneously with major resection in PVE-OSH patients and as separate stages in TSH-PVE and ALPPS patients) and details of the major resection procedure and postoperative outcomes

	PVE-OSH (*n* = 113)	TSH-PVE (*n* = 48)	ALPPS (*n* = 110)	*P*
**FLR clearing**				
1–2 local resections	86 (76.1)	25 (52.1)	75 (68.6)	<0.001*
3–4 local resections	16 (14.2)	10 (20.8)	23 (20.9)	–
≥5 local resections	1 (0.9)	9 (18.8)	4 (3.6)	–
1 additional ablation	5 (4.4)	2 (4.2)	10 (9.1)	0.274
≥2 additional ablations	4 (3.5)	1 (2.1)	7 (6.4)	–
No resection				
Only ablation	3 (2.7)	4 (8.3)	7 (6.4)	0.252
Vanishing lesions	7 (6.2)	0 (0.0)	1 (0.9)	0.027*
Complications				
None	12 (70.6)	41 (95.3)	44 (84.6)	0.177
Minor (I + II)	5 (29.4)	1 (2.3)	6 (11.6)	–
Major (IIIa/b + IV)	0 (0.0)	1 (2.3)	2 (3.8)	–
Death (V)	0 (0.0)	0 (0.0)	0 (0.0)	–
Operating time (min), median (i.q.r.)	244 (172–315)	145 (91–225)	246 (193–290)	<0.001*
Intraoperative bleeding (min), median (i.q.r.)	600 (350–1000)	200 (20–300)	600 (325–1050)	<0.001*
**Major resection**				
Right hepatectomy	69 (61.1)	38 (79.2)	49 (44.5)	<0.001*
Extended right hepatectomy	44 (38.9)	10 (20.8)	60 (54.5)	<0.001*
Left hepatectomy	0 (0.0)	0 (0.0)	1 (0.9)	0.480
Additional FLR resection/ablation		11 (22.9)	7 (6.4)	0.003*
Operating time (min), median (i.q.r.)	244 (172–303)	209 (128–304)	98 (67–155)	<0.001*
Intraoperative bleeding (min), median (i.q.r.)	500 (300–900)	700 (450–1250)	225 (50–683)	<0.001*
Complications				
None	44 (38.9)	21 (43.8)	42 (38.2)	0.885
Minor (I + II)	26 (23.0)	11 (22.9)	21 (19.1)	–
Major (IIIa/b + IV)	38 (33.6)	15 (31.3)	40 (36.4)	–
Death (V)	5 (4.4)	1 (2.1)	7 (6.4)	–
90-day mortality	7 (6.2)	1 (2.1)	10 (9.1)	0.258
PHLF				
None	91 (80.5)	42 (87.5)	84 (76.4)	0.437
A	6 (5.3)	2 (4.2)	12 (10.9)	–
B	6 (5.3)	2 (4.2)	8 (7.3)	–
C	10 (8.8)	2 (4.2)	6 (5.5)	–
Bile leak				
None	101 (89.4)	38 (79.2)	86 (78.2)	0.960
A	1 (0.9)	3 (6.3)	10 (9.1)	–
B	11 (9.7)	6 (12.5)	12 (10.9)	–
C	0 (0.0)	1 (2.1)	2 (1.8)	–
R1	47 (41.6)	17 (35.4)	38 (34.5)	0.521
Liver recurrence <6 months	22 (19.5)	14 (29.2)	34 (30.9)	0.126

Values are *n* (%) unless otherwise indicated. In the FLR clearing section, complications, operating time, and intraoperative bleeding in the PVE-OSH group are shown for patients who required rescue ALPPS. *Statistically significant. FLR, future liver remnant; PVE-OSH, portal vein embolization with no prior tumour clearance in the FLR followed by a one-stage hepatectomy; TSH-PVE, tumour clearance in the FLR followed by portal vein embolization (where TSH stands for two-stage hepatectomy); ALPPS, associating liver partition and portal vein ligation for staged hepatectomy; i.q.r., interquartile range; PHLF, post-hepatectomy liver failure; R1, tumour cells <1 mm from the resection plane.

### Major liver resection

Right hepatectomy was performed more often for PVE-OSH patients (61%) and TSH-PVE patients (79%) compared with ALPPS patients (45%) (*P* < 0.001) (*Table 2*). Furthermore, an additional resection or ablation during the second step was necessary for 23% of TSH-PVE patients and 6% of ALPPS patients (*P* = 0.003) (*Table 2*).

### Postoperative outcomes

Overall postoperative complications and liver-specific complications such as PHLF and bile leakage were comparable between the three groups (*Table 2*). Also, 90-day mortality was not significantly different, but ALPPS patients had the highest rate (9%).

### Liver recurrence-free survival, disease-free survival, and overall survival

**Fig. 2:**
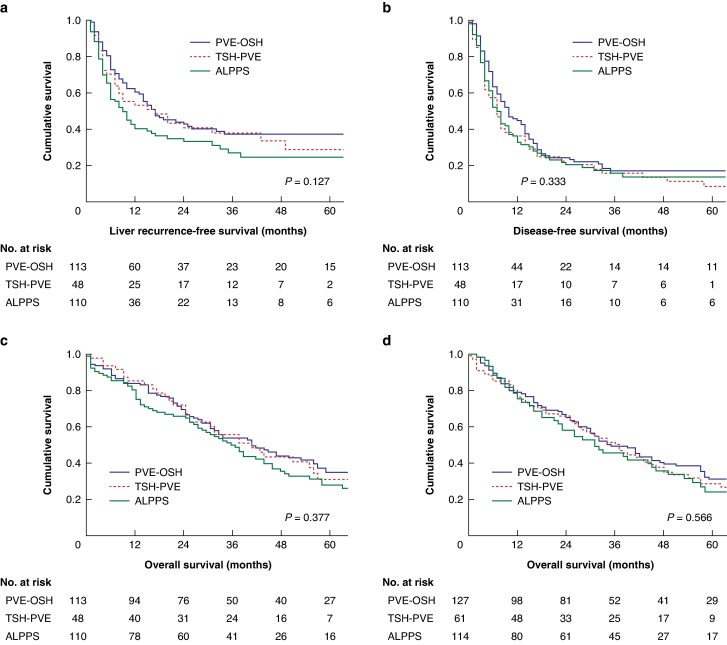
Survival **a** Liver recurrence-free survival. **b** Disease-free survival. **c** Overall survival from major resection. **d** Overall survival from first intervention with all patients included. PVE-OSH, portal vein embolization with no prior tumour clearance in the future liver remnant followed by a one-stage hepatectomy; TSH-PVE, tumour clearance in the future liver remnant followed by portal vein embolization (where TSH stands for two-stage hepatectomy); ALPPS, associating liver partition and portal vein ligation for staged hepatectomy.

The liver recurrence-free survival outcomes are illustrated in *Fig. 2a*, demonstrating no significant difference among the groups. The 5-year liver recurrence-free survival was 37% for PVE-OSH patients, 29% for TSH-PVE patients, and 25% for ALPPS patients (*P* = 0.127) (*Fig. 2a*). No significant differences were observed between the groups in terms of disease-free survival and overall survival (*Fig. 2b*,*c*). *Figure 2d* shows the overall survival measured from the first intervention, including patients who did not undergo major resection. A Cox regression analysis was performed to identify factors associated with liver recurrence-free survival. The analysis revealed that ALPPS and the total number of lesions were independent risk factors for liver recurrence-free survival (*Table 3*).

**Table 3:** Cox proportional hazard regression analysis of factors associated with liver recurrence-free survival

Parameter	Univariate analysis		Multivariable analysis	
	HR (95% c.i.)	*P*	HR (95% c.i.)	*P*
Age	0.99 (0.97,0.99)	0.035	0.99 (0.97,1.00)	0.083
Female	1.24 (0.87,1.75)	0.231	–	–
ECOG grade 0	Reference	–	–	–
ECOG grade 1	1.15 (0.83,1.60)	0.387	–	–
ECOG grade 2	0.82 (0.33,2.01)	0.656	–	–
Right colon	Reference	–	–	–
Left colon	1.23 (0.81,1.88)	0.338	–	–
Rectum	1.23 (0.80,1.88)	0.349	–	–
TSH-PVE	Reference	–	–	–
PVE-OSH	0.90 (0.58,1.38)	0.620	0.99 (0.62,1.58)	0.968
ALPPS	1.30 (0.84,2.01)	0.239	1.64 (1.00,2.68)	0.048
Metachronous	0.98 (0.76,1.27)	0.883	–	–
Number of lesions	1.03 (1.00,1.06)	0.065	1.04 (1.00,1.07	0.025
Largest lesion (mm)	1.00 (0.99,1.01)	0.535	–	–
Number of lesions in the FLR	1.03 (0.94,1.13)	0.498	–	–
Largest lesion in the FLR (mm)	1.01 (0.99,1.02)	0.332	–	–
Baseline sFLR (%)	1.02 (1.00,1.05)	0.064	1.02 (0.99,1.04)	0.191
Pre-resection sFLR (%)	1.01 (1.00,1.03)	0.186	0.99 (0.97,1.02)	0.875
Hypertrophy time (weeks)	1.00 (0.98,1.03)	0.768	–	–
Absolute growth (ml)	1.00 (0.99,1.00)	0.764	–	–
Degree of hypertrophy (%)	1.00 (0.98,1.03)	0.919	–	–
KGR (%/week)	1.01 (0.99,1.03)	0.427	–	–
R1	1.27 (0.92,1.78)	0.144	1.37 (0.97,1.92)	0.074

ECOG PSS, Eastern Cooperative Oncology Group Performance Status Scale; TSH-PVE, tumour clearance in the future liver remnant followed by portal vein embolization (where TSH stands for two-stage hepatectomy); PVE-OSH, portal vein embolization with no prior tumour clearance in the future liver remnant followed by a one-stage hepatectomy; ALPPS, associating liver partition and portal vein ligation for staged hepatectomy; FLR, future liver remnant; sFLR, standardized future liver remnant; KGR, kinetic growth rate; R1, tumour cells <1 mm from the resection plane.

## Discussion

This study compares different surgical strategies for bilateral CRLM with a tumour burden in the FLR. In patients with bilateral CRLM that are not resectable with a one-stage parenchymal-sparing resection, a two-stage procedure is often selected as the primary resection strategy^28,29^. In this study, an alternative approach is presented, consisting of an upfront PVE without prior tumour clearance in the FLR, followed by a simultaneous major resection of the embolized lobe and local resections in the FLR (PVE-OSH). This one-stage technique is a safe alternative to two-stage procedures such as TSH-PVE and ALPPS. In the present study, the PVE-OSH approach resulted in comparable or preferable outcomes with respect to liver recurrence-free survival, morbidity, mortality, and overall survival.

Compared with unilateral disease, bilateral CRLM present a more challenging clinical scenario due to higher morbidity and mortality^29–31^, an increased recurrence rate, and reduced survival^32,33^. Multiple resection strategies are often feasible and the optimal strategy remains to be elucidated. A strategy of one-stage parenchyma-sparing liver resection, with ultrasound-guided multiple resections and skeletonization of vessels, aims to reduce the risks of PHLF and morbidity, while maintaining oncological survival^34–36^. However, large comparative studies are lacking.

Milestones in regenerative liver surgery were set by Makuuchi *et al*.^37^ with the introduction of PVE and Adam *et al*.^38^ with the introduction of TSH. Since then, the concept of regenerative liver surgery has undergone continuous development, resulting in a variety of procedures^2,39^. The most common approach involves the clearance of the FLR during the initial procedure, followed by PVE and, after hypertrophy, major resection is performed. A recent multicentre study demonstrated a liver failure rate of 10% and a 90-day mortality rate of 4.5%^29^. Another study (conducted at the MD Anderson Cancer Center) revealed a completion rate of 64% and a 90-day mortality rate of 6% for TSH-PVE^40^. A comparison of these two studies with the present study reveals similar results of TSH-PVE. However, it is noteworthy that, in the present study, the PVE-OSH group had a completion rate of 89%.

The ALPPS procedure, known for its rapid hypertrophy, has been the subject of extensive debate. Initial concerns regarding its high morbidity and mortality rates have been partially mitigated through surgical modifications, patient selection, and accumulated experience^31,41^. Prospective and retrospective studies have not consistently demonstrated its inferiority to other surgical techniques, such as TSH^13,42–45^. The randomized LIGRO trial was the first and only trial to directly compare TSH and ALPPS, and it demonstrated comparable complication rates^13^ and superior long-term survival for ALPPS^46^. It is noteworthy that most participating centres from the LIGRO trial also participated in the present study and that some trial patients are also included in the present study.

The increased utilization of PVE has sparked concern regarding the potential for tumour growth during the regenerative process in CRLM^7,47^. Conversely, recent studies have demonstrated tumour stability during the regeneration process after PVE because of advancements in modern chemotherapy regimens^9–11^. Consequently, certain centres have adopted a PVE-OSH approach, omitting prior FLR clearance, followed by a one-stage major resection of the embolized lobe and local resections in the contralateral lobe. Spelt *et al*.^9^ reported on 23 patients with resected bilateral CRLM after PVE of the right liver lobe and tumour in the FLR, and there was no difference in tumour growth. Another study presented 33 cases with a positive response to chemotherapy and showed a good impact on tumour growth after PVE in both lobes^11^. A study conducted at the Memorial Sloan Kettering Cancer Center investigated the impact of chemotherapy on liver growth and tumour growth after PVE and concluded that chemotherapy does not inhibit FLR hypertrophy and may prevent cancer progression^10^; the study included nine patients who underwent hemihepatectomy and simultaneous contralateral local resections. However, none of these mentioned studies compared the efficacy of this procedure with that of other available resection strategies or investigated the associated morbidity. Also, all previous studies only involved a small number of patients, limiting the strength of the presented results. The advantages of the PVE-OSH approach lie in its ability to eliminate the need for two separate surgical interventions and to minimize the interval between chemotherapy and resection, because PVE can be performed during or shortly after chemotherapy. In contrast, for TSH-PVE patients, the time from end of chemotherapy or from first stage to completion was the longest. The extended interval without chemotherapy could be a contributing factor to the higher dropout rate during the hypertrophy process (21%) for the TSH-PVE patients. For all three groups, the primary reason for not undergoing major resection was tumour progression, underscoring the critical role of chemotherapy in this patient population. TSH-PVE and ALPPS procedures necessitated additional FLR resection or ablation during the second stage. This observation suggests that residual or undetectable lesions may have recurred during the hypertrophy. The PVE-OSH group had the highest number of vanishing lesions, although this finding requires cautious interpretation, as it may be attributable to registration bias in the other groups. Furthermore, the TSH-PVE group had the highest tumour load, resulting in a significant number of local resections in the FLR. This may be a contributing factor to the inferior outcomes observed in this group.

Conversely, ALPPS caused rapid hypertrophy and reduced time intervals to major resection, consequently yielding a minimal dropout rate. The recurrence rate was highest in the ALPPS group, a finding that aligns with prior studies^42,48^. This observation suggests that the dropouts in the TSH-PVE and PVE-OSH groups may be due to the selection process, indicating that these patients might have experienced early recurrence if they had undergone ALPPS. It appears that the selection process was similar, but at different time points.

The outcome of interest of this study was liver recurrence-free survival and, although a non-significant trend was observed, the long-term liver recurrence rates were comparable to overall survival rates, consistent with previous studies^41,43,46^. In the multivariable analysis, risk of liver recurrence was associated with a higher number of metastases and patients undergoing ALPPS.

A comparison of the three groups revealed similar complication rates. Although there was no statistical significance, ALPPS patients had a 90-day mortality rate of 9%, which is consistent with the findings of other ALPPS studies^31,43,49^. Nevertheless, the mortality rate observed for ALPPS appears to be rather high, highlighting the current debate on whether ALPPS should be used exclusively as a salvage strategy rather than a standard approach. In addition, the overall PHLF rate (any grade) was 20% for PVE-OSH patients, 13% for TSH-PVE patients, and 24% for ALPPS patents (only grade B/C: 14%, 8%, and 13% respectively). The elevated PHLF rate observed in the PVE-OSH group can possibly be attributed to inaccuracy of FLR measurements. For PVE-OSH, the volumetry of the left liver lobe necessitates tumour exclusion and a margin between the tumour and future resection line. Whether the estimated margin reflects the actual extent of resection may be doubted. In contrast, volumetry is quite feasible for TSH-PVE and ALPPS, given that volumetry is performed on a tumour-free liver lobe with clearly delineated anatomical landmarks.

This study has several limitations and drawbacks that must be considered. First, its retrospective design and slight, but present, differences in patient characteristics and tumour burdens. There might be a selection bias, in that TSH-PVE was preferably chosen for patients with a high tumour burden or complex location of metastases in the FLR. The study also had missing data associated with recurrence (such as nodal status, mutational status, and carcinoembryonic antigen levels). Furthermore, patients who underwent hepatic vein embolization were excluded to keep groups homogeneous and because the introduction of this method during the last years of the study interval would have resulted in a low number of patients.

## Supplementary Material

znaf198_Supplementary_Data

## Data Availability

The data supporting the findings of this study are available from the corresponding author upon reasonable request.

## References

[1] Heil J, Korenblik R, Heid F, Bechstein WO, Bemelmans M, Binkert C et al Preoperative portal vein or portal and hepatic vein embolization: DRAGON collaborative group analysis. Br J Surg 2021;108:834–842 33661306 10.1093/bjs/znaa149

[2] Heil J, Schiesser M, Schadde E. Current trends in regenerative liver surgery: novel clinical strategies and experimental approaches. Front Surg 2022;9:903825 36157407 10.3389/fsurg.2022.903825 PMC9491020

[3] Adam R, Kitano Y. Multidisciplinary approach of liver metastases from colorectal cancer. Ann Gastroenterol Surg 2019;3:50–56 30697610 10.1002/ags3.12227 PMC6345652

[4] de Graaf W, van Lienden KP, van den Esschert JW, Bennink RJ, van Gulik TM. Increase in future remnant liver function after preoperative portal vein embolization. Br J Surg 2011;98:825–834 21484773 10.1002/bjs.7456

[5] Huiskens J, Olthof PB, Van Der Stok EP, Bais T, Van Lienden KP, Moelker A et al Does portal vein embolization prior to liver resection influence the oncological outcomes—a propensity score matched comparison. Eur J Surg Oncol 2018;44:108–114 29126672 10.1016/j.ejso.2017.09.017

[6] Hoekstra LT, van Lienden KP, Doets A, Busch ORC, Gouma DJ, van Gulik TM. Tumor progression after preoperative portal vein embolization. Ann Surg 2012;256:812–818 23095626 10.1097/SLA.0b013e3182733f09

[7] Pamecha V, Levene A, Grillo F, Woodward N, Dhillon A, Davidson BR. Effect of portal vein embolisation on the growth rate of colorectal liver metastases. Br J Cancer 2009;100:617–622 19209170 10.1038/sj.bjc.6604872 PMC2653734

[8] de Graaf W, van den Esschert JW, van Lienden KP, van Gulik TM. Induction of tumor growth after preoperative portal vein embolization: is it a real problem? Ann Surg Oncol 2009;16:423–430 19050974 10.1245/s10434-008-0222-6

[9] Spelt L, Sparrelid E, Isaksson B, Andersson RG, Sturesson C. Tumour growth after portal vein embolization with pre-procedural chemotherapy for colorectal liver metastases. HPB 2015;17:529–535 25726854 10.1111/hpb.12397 PMC4430784

[10] Fischer C, Melstrom LG, Arnaoutakis D, Jarnagin W, Brown K, D’Angelica M et al Chemotherapy after portal vein embolization to protect against tumor growth during liver hypertrophy before hepatectomy. JAMA Surg 2013;148:1103–1108 24173207 10.1001/jamasurg.2013.2126

[11] Pommier R, Ronot M, Cauchy F, Gaujoux S, Fuks D, Faivre S et al Colorectal liver metastases growth in the embolized and non-embolized liver after portal vein embolization: influence of initial response to induction chemotherapy. Ann Surg Oncol 2014;21:3077–3083 24743912 10.1245/s10434-014-3700-z

[12] Brüning R, Schneider M, Tiede M, Wohlmuth P, Stavrou G, von Hahn T et al Ipsilateral access portal venous embolization (PVE) for preoperative hypertrophy exhibits low complication rates in Clavien-Dindo and CIRSE scales. CVIR Endovasc 2021;4:41 33999299 10.1186/s42155-021-00227-5 PMC8128945

[13] Sandström P, Røsok BI, Sparrelid E, Larsen PN, Larsson AL, Lindell G et al ALPPS improves resectability compared with conventional two-stage hepatectomy in patients with advanced colorectal liver metastasis: results from a Scandinavian multicenter randomized controlled trial (LIGRO trial). Ann Surg 2018;267:833–840 28902669 10.1097/SLA.0000000000002511 PMC5916470

[14] Røsok BI, Björnsson B, Sparrelid E, Hasselgren K, Pomianowska E, Gasslander T et al Scandinavian multicenter study on the safety and feasibility of the associating liver partition and portal vein ligation for staged hepatectomy procedure. Surgery 2016;159:1279–1286 26606881 10.1016/j.surg.2015.10.004

[15] Björnsson B, Hasselgren K, Røsok B, Larsen PN, Urdzik J, Schultz NA et al Segment 4 occlusion in portal vein embolization increase future liver remnant hypertrophy—a Scandinavian cohort study. International Journal of Surgery 2020;75:60–65 32001330 10.1016/j.ijsu.2020.01.129

[16] Carling U, Røsok B, Berger S, Fretland ÅA, Dorenberg E. Portal vein embolization using *N*-butyl cyanoacrylate-glue: what impact does a central vascular plug have? Cardiovasc Intervent Radiol 2022;45:450–458 34907454 10.1007/s00270-021-03014-w PMC8940786

[17] Vauthey J-N, Abdalla EK, Doherty DA, Gertsch P, Fenstermacher MJ, Loyer EM et al Body surface area and body weight predict total liver volume in Western adults. Liver Transpl 2002;8:233–240 11910568 10.1053/jlts.2002.31654

[18] Vauthey JN, Chaoui A, Do KA, Bilimoria MM, Fenstermacher MJ, Charnsangavej C et al Standardized measurement of the future liver remnant prior to extended liver resection: methodology and clinical associations. Surgery 2000;127:512–519 10819059 10.1067/msy.2000.105294

[19] Oken MM, Creech RH, Tormey DC, Horton J, Davis TE, McFadden ET et al Toxicity and response criteria of the Eastern Cooperative Oncology Group. Am J Clin Oncol 1982;5:649–655 7165009

[20] Siriwardena AK, Serrablo A, Fretland ÅA, Wigmore SJ, Ramia-Angel JM, Malik HZ et al Multisocietal European consensus on the terminology, diagnosis, and management of patients with synchronous colorectal cancer and liver metastases: an E-AHPBA consensus in partnership with ESSO, ESCP, ESGAR, and CIRSE. Br J Surg 2023;110:1161–1170 37442562 10.1093/bjs/znad124 PMC10416695

[21] Eisenhauer EA, Therasse P, Bogaerts J, Schwartz LH, Sargent D, Ford R et al New response evaluation criteria in solid tumours: revised RECIST guideline (version 1.1). Eur J Cancer 2009;45:228–247 19097774 10.1016/j.ejca.2008.10.026

[22] Shindoh J, Truty MJ, Aloia TA, Curley SA, Zimmitti G, Huang SY et al Kinetic growth rate after portal vein embolization predicts posthepatectomy outcomes: toward zero liver-related mortality in patients with colorectal liver metastases and small future liver remnant. J Am Coll Surg 2013;216:201–209 23219349 10.1016/j.jamcollsurg.2012.10.018 PMC3632508

[23] Sparrelid E, Gilg S, Brismar TB, Lundell L, Isaksson B. Rescue ALPPS is efficient and safe after failed portal vein occlusion in patients with colorectal liver metastases. Langenbecks Arch Surg 2017;402:69–75 27761713 10.1007/s00423-016-1524-y PMC5309264

[24] Clavien PA, Barkun J, de Oliveira ML, Vauthey JN, Dindo D, Schulick RD et al The Clavien-Dindo classification of surgical complications: five-year experience. Ann Surg 2009;250:187–196 19638912 10.1097/SLA.0b013e3181b13ca2

[25] Dindo D, Demartines N, Clavien P-A. Classification of surgical complications: a new proposal with evaluation in a cohort of 6336 patients and results of a survey. Ann Surg 2004;240:205–213 15273542 10.1097/01.sla.0000133083.54934.ae PMC1360123

[26] Rahbari NN, Garden OJ, Padbury R, Brooke-Smith M, Crawford M, Adam R et al Posthepatectomy liver failure: a definition and grading by the International Study Group of Liver Surgery (ISGLS). Surgery 2011;149:713–724 21236455 10.1016/j.surg.2010.10.001

[27] Koch M, Garden OJ, Padbury R, Rahbari NN, Adam R, Capussotti L et al Bile leakage after hepatobiliary and pancreatic surgery: a definition and grading of severity by the International Study Group of Liver Surgery. Surgery 2011;149:680–688 21316725 10.1016/j.surg.2010.12.002

[28] Dasari BVM, Raptis D, Syn N, Serrablo A, Ramia JM, Laurenzi A et al Development and validation of a novel risk score to predict overall survival following surgical clearance of bilobar colorectal liver metastases. BJS Open 2023;7:zrad085 37738617 10.1093/bjsopen/zrad085 PMC10516618

[29] Chavez MI, Gholami S, Kim BJ, Margonis GA, Ethun CG, Tsai S et al Two-stage hepatectomy for bilateral colorectal liver metastases: a multi-institutional analysis. Ann Surg Oncol 2021;28:1457–1465 33393036 10.1245/s10434-020-09459-6 PMC8385606

[30] Reese T, Gilg S, Böcker J, Wagner KC, Vali M, Engstrand J et al Impact of the future liver remnant volume before major hepatectomy. Eur J Surg Oncol 2024;50:108660 39243696 10.1016/j.ejso.2024.108660

[31] Linecker M, Kuemmerli C, Kambakamba P, Schlegel A, Muiesan P, Capobianco I et al Performance validation of the ALPPS risk model. HPB 2018;21:711–721 30477898 10.1016/j.hpb.2018.10.003

[32] Tsim N, Healey AJ, Frampton AE, Habib NA, Bansi DS, Wasan H et al Two-stage resection for bilobar colorectal liver metastases: R0 resection is the key. Ann Surg Oncol 2011;18:1939–1946 21298352 10.1245/s10434-010-1533-y

[33] Giuliante F, Viganò L, De Rose AM, Mirza DF, Lapointe R, Kaiser G et al Liver-first approach for synchronous colorectal metastases: analysis of 7360 patients from the LiverMetSurvey registry. Ann Surg Oncol 2021;28:8198–8208 34212254 10.1245/s10434-021-10220-w PMC8590998

[34] Torzilli G, Procopio F, Botea F, Marconi M, Del Fabbro D, Donadon M et al One-stage ultrasonographically guided hepatectomy for multiple bilobar colorectal metastases: a feasible and effective alternative to the 2-stage approach. Surgery 2009;146:60–71 19541011 10.1016/j.surg.2009.02.017

[35] Torzilli G, McCormack L, Pawlik T. Parenchyma-sparing liver resections. Int J Surg 2020;82:192–197 32335245 10.1016/j.ijsu.2020.04.047

[36] Torzilli G, Serenari M, Viganò L, Cimino M, Benini C, Massani M et al Outcomes of enhanced one-stage ultrasound-guided hepatectomy for bilobar colorectal liver metastases compared to those of ALPPS: a multicenter case-match analysis. HPB 2019;21:1411–1418 31078424 10.1016/j.hpb.2019.04.001

[37] Makuuchi M, Thai BL, Takayasu K, Takayama T, Kosuge T, Gunvén P et al Preoperative portal embolization to increase safety of major hepatectomy for hilar bile duct carcinoma: a preliminary report. Surgery 1990;107:521–527 2333592

[38] Adam R, Laurent A, Azoulay D, Castaing D, Bismuth H. Two-stage hepatectomy: a planned strategy to treat irresectable liver tumors. Ann Surg 2000;232:777–785 11088072 10.1097/00000658-200012000-00006 PMC1421270

[39] Imai K, Adam R, Baba H. How to increase the resectability of initially unresectable colorectal liver metastases: a surgical perspective. Ann Gastroent Surg 2019;3:476–486 10.1002/ags3.12276 PMC6749948 31549007

[40] Huang SY, Aloia TA, Shindoh J, Ensor J, Shaw CM, Loyer EM et al Efficacy and safety of portal vein embolization for two-stage hepatectomy in patients with colorectal liver metastasis. J Vasc Interv Radiol 2014;25:608–617 24315549 10.1016/j.jvir.2013.10.028

[41] Petrowsky H, Linecker M, Raptis DA, Kuemmerli C, Fritsch R, Kirimker OE et al First long-term oncologic results of the ALPPS procedure in a large cohort of patients with colorectal liver metastases. Ann Surg 2020;272:793–800 32833765 10.1097/SLA.0000000000004330

[42] Adam R, Imai K, Castro Benitez C, Allard M-A, Vibert E, Sa Cunha A et al Outcome after associating liver partition and portal vein ligation for staged hepatectomy and conventional two-stage hepatectomy for colorectal liver metastases. Br J Surg 2016;103:1521–1529 27517369 10.1002/bjs.10256

[43] Ratti F, Schadde E, Masetti M, Massani M, Zanello M, Serenari M et al Strategies to increase the resectability of patients with colorectal liver metastases: a multi-center case-match analysis of ALPPS and conventional two-stage hepatectomy. Ann Surg Oncol 2015;22:1933–1942 25564160 10.1245/s10434-014-4291-4

[44] Moris D, Ronnekleiv-Kelly S, Kostakis ID, Tsilimigras DI, Beal EW, Papalampros A et al Operative results and oncologic outcomes of associating liver partition and portal vein ligation for staged hepatectomy (ALPPS) versus two-stage hepatectomy (TSH) in patients with unresectable colorectal liver metastases: a systematic review and meta-analysis. World J Surg 2018;42:806–815 28798996 10.1007/s00268-017-4181-6

[45] Kambakamba P, Linecker M, Alvarez FA, Samaras P, Reiner CS, Raptis DA et al Short chemotherapy-free interval improves oncological outcome in patients undergoing two-stage hepatectomy for colorectal liver metastases. Ann Surg Oncol 2016;23:3915–3923 27431413 10.1245/s10434-016-5419-5

[46] Hasselgren K, Røsok BI, Larsen PN, Sparrelid E, Lindell G, Schultz NA et al ALPPS improves survival compared with TSH in patients affected of CRLM: survival analysis from the randomized controlled trial LIGRO. Ann Surg 2021;273:442–448 32049675 10.1097/SLA.0000000000003701

[47] Pamecha V, Glantzounis G, Davies N, Fusai G, Sharma D, Davidson B. Long-term survival and disease recurrence following portal vein embolisation prior to major hepatectomy for colorectal metastases. Ann Surg Oncol 2009;16:1202–1207 19130138 10.1245/s10434-008-0269-4

[48] Oldhafer KJ, Donati M, Jenner RM, Stang A, Stavrou GA. ALPPS for patients with colorectal liver metastases: effective liver hypertrophy, but early tumor recurrence. World J Surg 2014;38:1504–1509 24326456 10.1007/s00268-013-2401-2

[49] Reese T, Galavics C, Schneider M, Brüning R, Oldhafer KJ. Sarcopenia influences the kinetic growth rate after ALPPS. Surgery 2022;172:926–932 35606183 10.1016/j.surg.2022.04.022

