# Supplementary material for: Oncological safety of portal vein embolization without prior tumour clearance in the future liver remnant followed by one-stage hepatectomy for bilateral colorectal liver metastases
Source: Br J Surg. 2025 Sep 22;112(9):znaf198. doi: 10.1093/bjs/znaf198 (PMC12452274; doi:10.1093/bjs/znaf198)
Supplement: znaf198_Supplementary_Data [file znaf198_supplementary_data.docx]

**Oncological safety of portal vein embolization without prior tumour clearance in the future liver remnant followed by one-stage hepatectomy for bilateral colorectal liver metastases**

Tim Reese1, Dennis Björk2, Anne Madsi Holmen Longva3, Kristian Schaumburg Kiim4, Maximilian Evers5, Peter Nørgaard Larsen4, Nicolai Aagaard Schultz4, Bård Ingvald Røsok3, Ulrik Carling5, Fredrik Holmquist7, Gert Lindell7, Per Sandström2, Jörg Böcker6, Stefan Gilg1, Jennie Engstrand1, Christian Sturesson1, Karl J. Oldhafer6, Bergthor Björnsson2 and Ernesto Sparrelid1

1 Division of Surgery and Oncology, Department of Clinical Science, Intervention and Technology, Karolinska Institutet, Karolinska University Hospital, Stockholm, Sweden.

2 Department of Surgery in Linköping and Department of Biomedical and Clinical Sciences, Linköping University, Linköping, Sweden.

3 Department of Hepato-Pancreato-Biliary Surgery, Oslo University Hospital, Oslo, Norway.

4 Department of Surgical Gastroenterology and Transplantation, Rigshospitalet, University of Copenhagen, Denmark.

5 Department of Radiology, Rigshospitalet, Oslo University Hospital, Oslo, Norway

6 Department of Surgery, Division of Hepatobiliary and Pancreatic Surgery, Asklepios Hospital Barmbek, Hamburg, Germany.

7 Department of Surgery, Skåne University Hospital, Lund, Sweden.

**Corresponding author: Tim Reese (MD), Division of Surgery and Oncology, Department of Clinical Science, Intervention and Technology, Karolinska Institutet, Karolinska University Hospital, Hälsovägen 13, 141 57 Stockholm, Sweden.** [**tim.reese@ki.se**](mailto:tim.reese@ki.se)

**Supplementary Materials - Index**

| **Supplementary Figures and Tables** |  |
| --- | --- |
| Supplementary Figure 1 - Flow chart of patient inclusion within the different groups and total number and percentage of patient not undergoing resection. | *page 2* |
| Supplementary Table 1 – Liver volume and volume of the FLR before and after hypertrophy and hypertrophy failures with change to rescue ALPPS | *page 3* |
|  |  |

**Supplementary Figures and Tables**

**
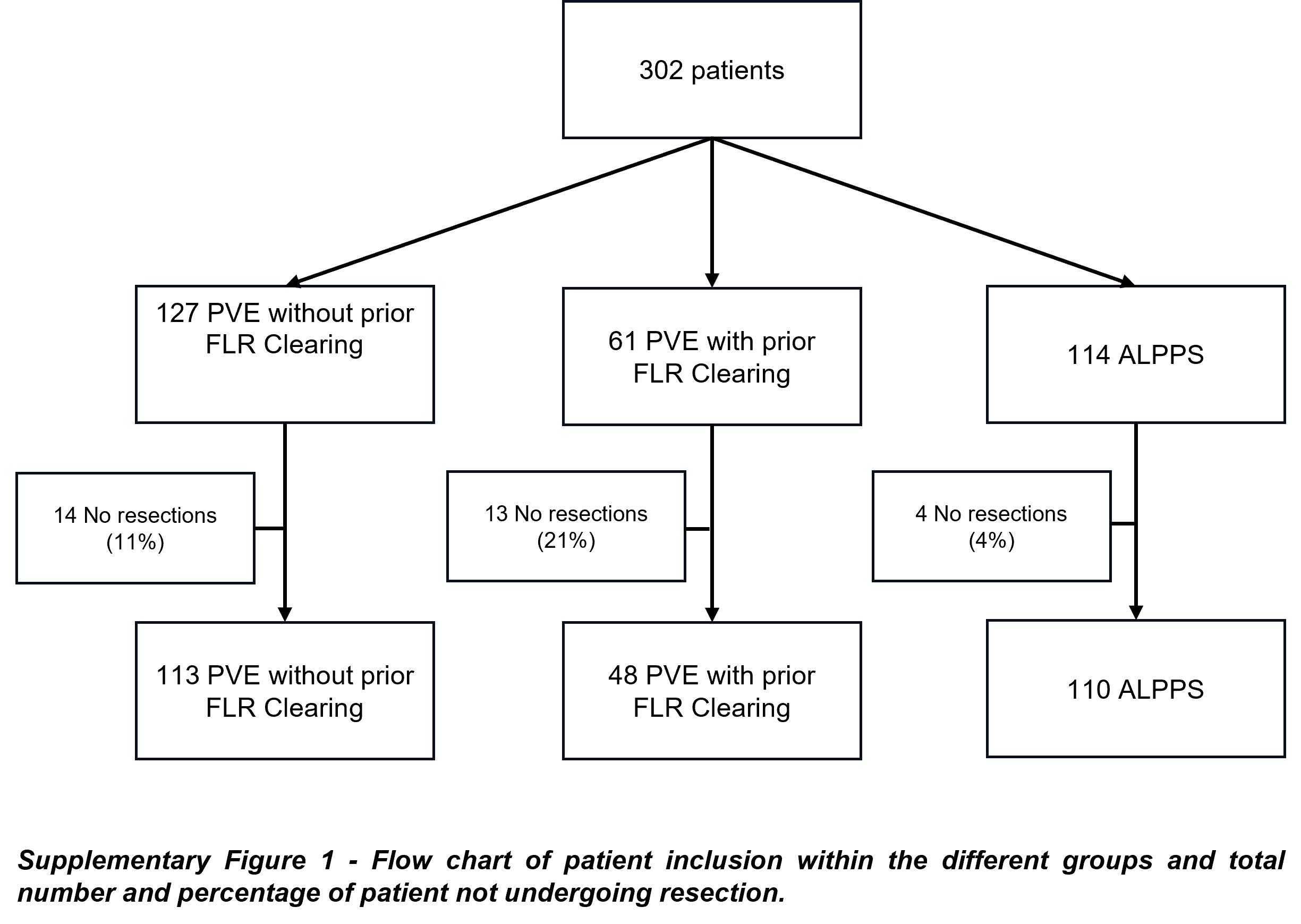
**

| ***Supplementary Table 1 – Detailed description for not proceeding with the completion resection*** | | | | | | |
| --- | --- | --- | --- | --- | --- | --- |
|  | **PVE-OSH  (n=14)** | | **TSH-PVE (n=13)** | | **ALPPS (n=4)** | |
| Overall progression (Extrahepatic + liver), n (%) | 2 | (14) | 1 | (8) | 3 | (75) |
| Extrahepatic progression only, n (%) | 6 | (43) | 3 | (23) |  |  |
| Progression in FLR only, n (%) | 2 | (14) | 3 | (23) |  |  |
| Progression in FLR + non-FLR, n (%) | 4 | (29) | 5 | (38) | 1 | (25) |
| Other: Portal hypertension, n (%) |  |  | 1 | (8) |  |  |
| Abbreviation: FLR (future liver remnant), sFLR (standardized future liver remnant), ALPPS (Associating Liver Partition and Portal vein Ligation for Staged hepatectomy) | | | | | | |

| ***Supplementary Table 2 – Liver volume and volume of the FLR before and after hypertrophy and hypertrophy failures with change to rescue ALPPS*** | | | | | | | |
| --- | --- | --- | --- | --- | --- | --- | --- |
|  | **PVE-OSH  (n=113)** | | **TSH-PVE (n=48)** | | **ALPPS (n=110)** | | **p-value** |
| BSA, median (IQR) | 2.0 | (1.8-2.1) | 2.0 | (1.8-2.1) | 1.9 | (1.8-2.1) | 0.846 |
| TELV, median (IQR) | 1696 | (1496-1864) | 1692 | (1455-1905) | 1653 | (1484-2876) | 0.846 |
|  |  |  |  |  |  |  |  |
| Baseline FLR (ml), median (IQR) | 389 | (320-478) | 379 | (303-468) | 387 | (314-485) | 0.939 |
| Baseline sFLR (%), median (IQR) | 23.0 | (19.7-2.8) | 24.6 | (18.9-28.2) | 24.8 | (20.3-29.5) | 0.581 |
| Preresection FLR (ml), median (IQR) | 600 | (519-711) | 581 | (50-675) | 610 | (510-768) | 0.293 |
| Preresection sFLR (%), median (IQR) | 36.1 | (31.3-40.6) | 34.0 | (30.7-41.9) | 37.6 | (32.7-45.0) | 0.050 |
|  |  |  |  |  |  |  |  |
| Hypertrophy Time (weeks), median (IQR) | 3.9 | (3.0-4.9) | 5.1 | (3.9-8.3) | 1.1 | (1.0-2.0) | **<0.001** |
| Absolut Growth (ml), median (IQR) | 207 | (132-283) | 197 | (116-319) | 214 | (151-345) | 0.131 |
| Degree of hypertrophy (%), median (IQR) | 11.8 | (7.8-17.4) | 11.7 | (7.3-16.8) | 14.3 | (9.9-19.0) | 0.072 |
| KGR (%/week), median (IQR) | 2.8 | (2.0-4.2) | 2.3 | (1.8-3.7) | 10.7 | (4.8-16.0) | **<0.001** |
|  |  |  |  |  |  |  |  |
| Rescue-ALPPS, n (%) | 17 | (15) | 12 | (35) |  |  | 0.133 |
|  |  |  |  |  |  |  |  |
| First intervention till major resection (weeks), median (IQR) | 6 | (5-8) | 9 | (7-13) | 1.4 | (1-3) | **<0.001** |
|  |  |  |  |  |  |  |  |
| Abbreviation: BSA (Body Surface Area), TELV (total estimated liver volume), FLR (future liver remnant), sFLR (standardized future liver remnant), KGR (Kinetic growth rate), ALPPS (Associating Liver Partition and Portal vein Ligation for Staged hepatectomy) | | | | | | | |
